# Diversity and composition of the bacterial communities associated with the Australian spittlebugs *Bathyllus albicinctus* and *Philagra parva* (Hemiptera: Aphrophoridae)

**DOI:** 10.1371/journal.pone.0311938

**Published:** 2024-10-10

**Authors:** Francesco Martoni, Lea Rako, Duncan Jaroslow, Caitlin Selleck, Pragya Kant, Narelle Nancarrow, Mark J. Blacket

**Affiliations:** 1 Agriculture Victoria, AgriBio Centre, Bundoora, Victoria, Australia; 2 Agriculture Victoria, Grains Innovation Park, Horsham, Victoria, Australia; Lund University, SWEDEN

## Abstract

Spittlebugs and froghoppers (Hemiptera: Cercopoidea) are insects feeding on xylem, which potentially can cause significant economic damage worldwide by transmitting plant pathogenic bacteria such as *Xylella fastidiosa*. Australia and New Zealand are currently free from *X*. *fastidiosa*, but they are home to at least 45 native spittlebug species. Among these, the Australian natives *Bathyllus albicinctus* (Erichson, 1842) and *Philagra parva* (Donovan, 1805) are particularly widespread and can be found across southern and eastern Australia, with *B*. *albicinctus* also in New Zealand. The potential that both species might be capable of vectoring *Xylella fastidiosa* poses a substantial biosecurity risk if the bacterium were to invade these regions. In this study, we examined 87 spittlebug nymphs collected across 12 different host plant species, in five locations in Victoria, Australia. Our objective was to explore the factors influencing bacterial communities within and between these widespread spittlebug species, considering geographic location, insect phylogenetics, and host plant associations. We employed COI barcoding to assess insect genetic variation and 16S high throughput sequencing (HTS) metabarcoding to analyse bacterial microbiome diversity across various host plants. Our findings revealed minimal genetic divergence among spittlebug individuals in the same species, highlighting conspecificity despite conspicuous morphological divergences. On the other hand, we recorded significant variation in bacterial communities harboured by *Bathyllus albicinctus* nymphs feeding on different plants, even when these were collected within close proximity to each other. Therefore, host plant association appeared to shape the bacterial communities of spittlebugs more than insect genetic divergence or geographical location. These diverse bacterial communities could potentially facilitate transmission of plant pathogenic bacteria, underscoring the risk of widespread transmission among numerous plant hosts through insect-plant interactions. This study emphasizes the critical need to understand these complex interactions, particularly in the context of biosecurity.

## Introduction

The superfamily Cercopoidea (Hemiptera: Auchenorrhyncha) is composed of more than 2600 described species worldwide, across more than 360 genera and five families [1,2]. Many Cercopoidea are known to be associated with nitrogen‐fixing angiosperms, with host plant differences associated with each insect family. For example, cercopids are generally associated with nitrogen‐fixing grasses, clastopterids with actinorhizal plants, and aphrophorids with legumes [3]. In general, most spittlebugs are known to be associated with a variety of host plants but generally play a limited role in plant‐pathogen transmission. For example, spittlebugs do not transmit plant viral pathogens (e.g., [2,4]), and are generally poor vectors of bacterial diseases since bacterial pathogens are usually phloem‐limited [5]. A notable exception to this rule is the xylem-blocking bacterium *Xylella fastidiosa* [6], commonly vectored by the meadow spittlebug *Philaenus spumarius* (Linnaeus, 1758) (Hemiptera: Aphrophoridae), causing economically important diseases such as the olive quick decline syndrome [7].

Australia is home to at least forty described cercopoid species, belonging to at least sixteen genera and three families, including taxa with distributions limited to Australian territories such as Norfolk, Lord Howe, and Christmas Islands [8]. Most of these species are endemic to Australia, with some native species also present in Papua New Guinea, New Zealand and Indonesia [8]. These are known to be associated with a variety of host plants, including native and introduced species. Notably, no Australian cercopoid species is currently known to be a pest, and no exotic species are known to be present in the country. On the other hand, the exotic *Philaenus spumarius* was introduced to New Zealand around 1960 [9] and poses the main cercopoid threat to Australian plant health due to its potential to vector deadly plant pathogens.

Insect-bacteria associations have been studied broadly, with a particular focus on hemipteran insects hosting bacterial symbionts required for the biosynthesis of nutrients [10–14] or vectoring plant pathogens of economic importance [15–18]. The obligate endosymbionts, also known as ‘primary endosymbionts’, supplement the insect hosts’ diets through the provision of essential nutrients such as amino acids, and are vertically transmitted from the parental line to the offspring across many hemipteran insects, including aphids, psyllids and white flies [10,19–21]. The facultative endosymbionts, or ‘secondary endosymbionts’, are not essential for insect host survival and one or more secondary endosymbionts of the same or different supergroups may provide advantages to their insect hosts [22,23].

This is also the case for hemipteran insects of the superfamily Cercopoidea, for which two obligate symbionts were originally reported: *Candidatus* Sulcia muelleri and *Ca*. Zinderia insecticola [24]. While *Ca*. Sulcia species are common primary symbionts across other hemipteran groups, such as cicadas and leafhoppers, *Ca*. Zinderia insecticola was first described in spittlebugs [24]. However, some spittlebugs, including *P*. *spumarius* have switched one of their obligate symbionts, *Ca*. Zinderia insecticola, with a *Sodalis-*like species [25].

As the main cercopoid vector of *Xylella fastidiosa*, researchers worldwide have recently focused their attention on *P*. *spumarius* and have been studying its microbial associations more closely [7,25–27]. However, very little is known about the microbial communities harboured by other spittlebugs and what factors may impact their diversity and composition. Deeper knowledge of the bacterial diversity and community composition of phylogenetically related spittlebugs may provide an insight into bacterial transmission across populations as well as improving our understanding of the role different bacteria may play within the insect host. Indeed, recent studies have shown that other non-symbiotic bacteria, such as the taxa composing the gut microbiota, may play a pivotal role in insect-plant interactions [28–31].

Here we analysed the bacterial communities across 14 populations belonging to two widespread species of spittlebug—*Bathyllus albicinctus* and *Philagra parva* (both family Aphrophoridae)—collected from 12 host plants in Victoria, Australia. We compared bacterial communities associated with these insects across different spittlebug populations, different host plants and different geographic locations. The main aims of this work were to 1) explore the bacterial diversity of these Australian spittlebug species and 2) compare their obligate symbionts with those of other spittlebugs, including the main cercopoid vector of *Xylella*. This could provide potential insights into insect-plant specificity as well as on symbiont evolution. Additionally, we wanted to determine whether factors such as geographic location and host plant association contributed to shaping the bacterial communities associated with each insect population.

## Materials and methods

### Sample collection

A total of six adult and 64 nymph specimens of *Bathyllus albicinctus* were collected from three different locations in Victoria and from at least 10 host plants (Table 1). Additionally, 17 nymph specimens of another aphrophorid insect, *Philagra parva*, were collected from three populations and two different locations to be used for comparison (Table 1).

**Table 1:** Aphrophorid populations collected for this study. The table includes spittlebug species, location, and host plant, as well as accession numbers for the 84 specimens sequenced.

Insect Species	Location	Host plant	Individuals	COI Acc. numbers
***Bathyllus albicinctus***	Bundoora, VIC	*Carpobrotus* sp.	2	PP946048-49 (2)
		*Dianella* sp.	2	PP946053-54 (2)
		*Myoporum* sp.	5	PP946042-47 (5)
		*Poa* sp.	3	PP946050-52 (3)
		*Westringia* sp.	9	PP946034-41 (9)
	Grampians, VIC	*Gonocarpus* sp.	5	PP946088-92 (5)
		*Calluna* sp.	6	PP946080-84 (5)
		*Olea* sp.	3	PP946085-87 (3)
	Horsham, VIC	Lamiaceae sp.	10	PP946024-33 (10)
			6	PP946065-70 (6)
		*Petroselinum* sp.	3	PP946077-79 (3)
		*Westringia* sp.	10	PP946014-23 (10)
			6	PP946071-76 (6)
***Philagra parva***	Coburg, VIC	*Acacia* sp.	7	PP946093-97 (5)
	Moolap, VIC	*Acacia* sp.	6	PP946055-60 (6)
		*Casuarina* sp.	4	PP946061-64 (4)

Samples were collected from plants in modified environments (i.e., gardens, roadside vegetation) associated with people. Most of the plants are considered ornamentals and no significant variation could be observed in climatic conditions, vegetation type or soil type across the geographic locations where sampling was conducted. Nymphs were generally found surrounded by water bubble excretions, termed ‘spittle’, and were collected using a fine brush to transfer them in a vial containing high grade (100%) ethanol. Adults were collected using aspirators or captured manually into a vial, then immediately killed using high grade ethanol. All samples were collected from private properties and did not require any collection permit.

### DNA extraction, amplification and sequencing

DNA was extracted from single individual insects using a DNeasy Blood and Tissue kit (Qiagen, Germany), in a dedicated DNA extraction room to limit the chances of contamination. A DNA extraction negative control was included for each DNA extraction batch. Individual insects were homogenised using two 3mm glass beads in individual Eppendorf 1.7mL tubes in a TissueLyser II (Qiagen, Germany) at 30Hz for 3 minute, and left overnight (~17 hours) at 56 ⁰C in a 1:9 solution of proteinase K and ATL buffer. The following day, DNA was extracted following the manufacturer’s instructions and re-eluted in 100 μL. The same DNA extract was used to amplify both insect and bacterial DNA.

**Fig 1:**
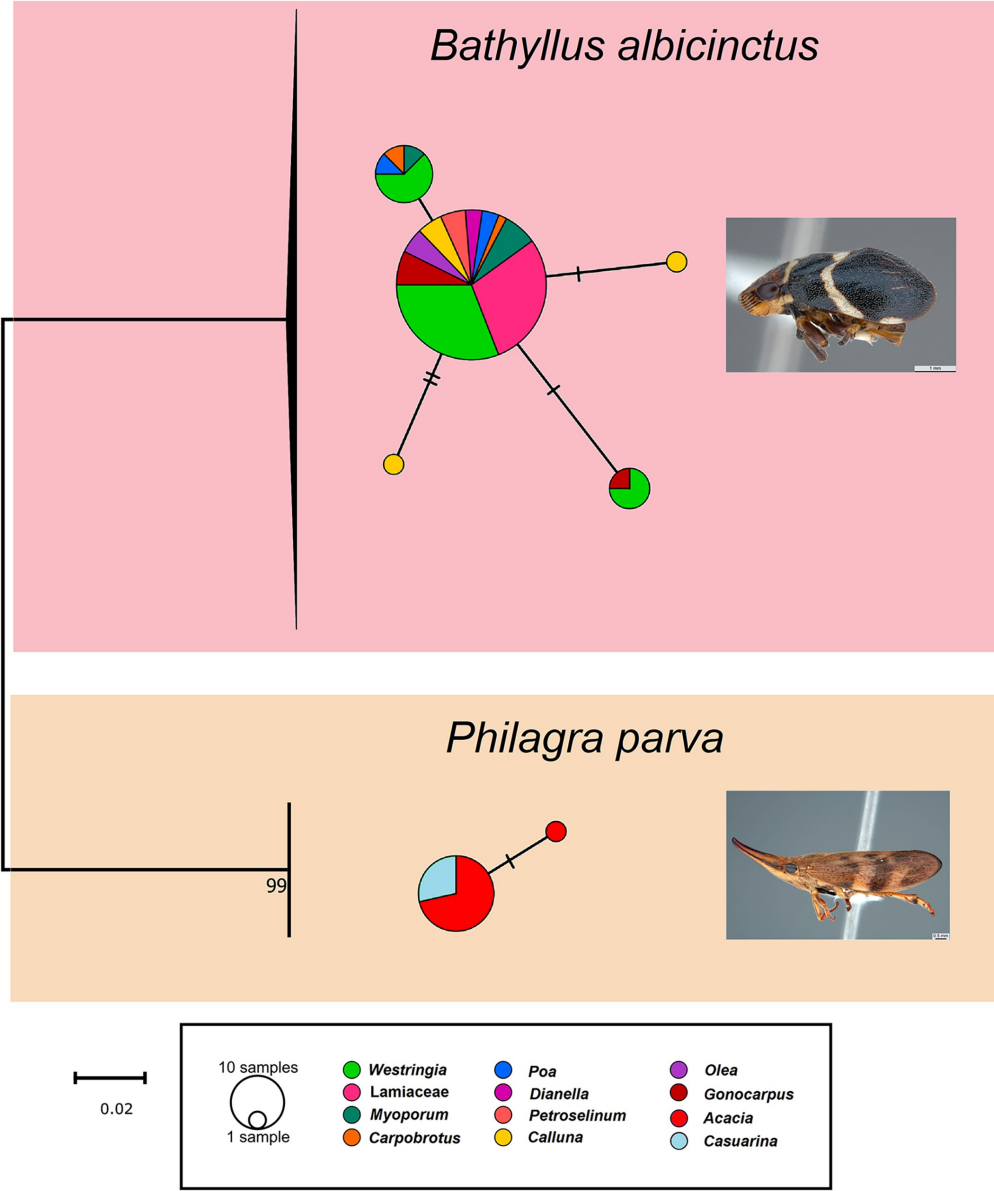
Haplotype network analysis of the 84 COI sequences of *Bathyllus albicinctus* and *Philagra parva* generated for this study. The network was obtained using 84 partial COI DNA sequences. The network is colour-coded based on the host plant from which each specimen was collected. The Neighbour Joining COI tree on the left was generated using 5,000 bootstrap replicates, and the scale bar represents a 2% genetic variation.

Due to the morphological similarity of aphrophorid nymphs, we used the DNA barcoding technique [32] to assess taxonomic identity and genetic variability across the different populations. A 642 bp fragment of the subunit I of the *cytochrome oxidase* (COI) mitochondrial gene was targeted using the newly designed cercopoid specific primers Cerco-F (5’- TTYGGDATTTGATCAGGAATAATTGG-3’) and Cerco-R (5’- GAATAAATGTTGRTATAAAATWGGRTC-3’). The PCRs were performed using the MyFi PCR kit (Meridian Biosciences, USA) in 25 μL volumes consisting of 14.7 μL of 100 ug/mL Bovine Serum Albumin (New England Biolabs, USA) diluted in ddH20, 5 μL of 5X MyFi Reaction Buffer, 1 μL of both the forward and reverse primers (10 μM), 0.8 μL of MyFi DNA Polymerase and 2.5 μL of DNA template. The following PCR cycling conditions were used: initial denaturation at 94 ⁰C for 2 minutes, followed by 30 cycles at 94 ⁰C for 30 seconds, 50 ⁰C for 45 seconds, 72 ⁰C for 45 seconds, and a final extension phase at 72 ⁰C for 7 minutes. Successful amplification was confirmed by running the PCR products on 1% Agarose gels for 30 minutes at 100V. Successful PCR amplicons were sequenced in both directions using an external provider (AGRF, Melbourne, Australia). Electropherograms were analysed using the software MEGA v11 [33]. Forward and reverse sequences were paired, and a consensus sequence was generated. A COI gene tree was generated using the Neighbour Joining algorithm and 5,000 bootstrap replicates (Fig 1). Curated sequences were submitted to the online repository GenBank with accession numbers PP946014-PP946097. The COI sequences obtained in this study were trimmed to be aligned together with the shortest sequence generated (542 bp) and the software PopART [34] was used to perform a Median Joining Network analysis [35] with ε = 0 (Fig 1).

For the analysis of the bacterial communities, we used a 16S metabarcoding approach modified from a similar work on psyllids (Hemiptera) [36]. Here a 253 bp fragment of the 16S gene was targeted using the primers Fwd515 (ACACTCTTTCCCTACACGACGCTCTTCCGATCT GTGCCAGCMGCCGCGGTAA)—Rev806 (GTGACTGGAGTTCAGACGTGTGCTCTTCCGATCT GGACTACHVGGGTWTCTAAT) [37], modified with partial Illumina adapters (underlined) and the following PCR cycling conditions: initial denaturation at 94 ⁰C for 2 minutes, followed by 35 cycles at 94 ⁰C for 30 seconds, 50 ⁰C for 45 seconds, 72 ⁰C for 45 seconds, and a final extension phase at 72 ⁰C for 7 minutes. Successful amplification was confirmed running the PCR products on 1% Agarose gels for 30 minutes at 100 V. PCR reactions were prepared in a dedicated room, working under a biological hood that had been cleaned with high grade (100%) ethanol and irradiated with UV light. PCRs were conducted in duplicate replicates, with negative controls for each PCR batch.

The remainder of the Illumina adapter sequences containing 8 bp unique dual indexes were then attached to each PCR product using a real-time PCR (rtPCR) with the Phusion High Fidelity PCR kit (Thermo Fisher Scientific, USA) following the same protocol presented elsewhere [38,39]. Each 50 μL reaction consisted of 32.5 μL of molecular grade water, 10 μL of 5X Phusion HF buffer, 1 μL of dNTP mix, 1 μL of 1/1000 SYBR Green I Mix, 0.5 μL of Phusion DNA polymerase, along with 4 μL of adapter primer mix with indexes unique to each sample (2.5 μM) and 1 μL of PCR product. The real-time PCR began with 30 seconds at 98°C, followed by 7 cycles of: 98°C for 10 seconds, 65°C for 30 seconds and 72°C for 30 seconds, with fluorescence measurement conducted in the 65°C and 72°C phases. The amplification curve was visually inspected in real time and stopped while still in the exponential phase to prevent over-amplification artefacts.

DNA concentrations of each library were normalised using the SequalPrep Normalization Plate Kit (Thermo Fisher Scientific, USA) following the manufacturer’s instructions, but re-eluting in 15 μL instead of 20 μL, then all samples were pooled together by target barcode. The DNA fragment size and concentration of the three pooled barcode libraries were measured using a High Sensitivity D1000 ScreenTape assay on a 2200 TapeStation (Agilent Technologies, USA). Pooled libraries were then cleaned and concentrated using the ProNex Chemistry Kit, following the manufacturer’s instructions to remove fragments > 350 base pairs.

Due to the number of samples, two final 16S libraries were diluted to a concentration of 7 pM, spiked with 15% PhiX and sequenced on two separate runs using V3 chemistry (2 x 250 bp reads) on an Illumina MiSeq system (Illumina, USA).

### Testing for high priority pest pathogens

Presence of certain plant pathogenic species can only be hypothesised, and not inferred, when based on analyses of partial 16S DNA sequences, which often cannot resolve taxa to species level. For these reasons, all samples included in this study were also tested for two high-priority plant pathogenic bacteria exotic to Australia: *Ralstonia solanacearum* species complex [40] and *Xylella fastidiosa*.

Primer sequences specific to *Ralstonia solanacearum* species complex are p759 (5′-GTCGCCGTCAACTCACTTTCC-3′) and p760 (5′-GTCGCCGTCAGCAATGCGGAATCG-3′) [41]. PCR conditions included an initial denaturation at 96°C for 5 minutes, followed by 30 cycles of denaturation at 94°C for 15 seconds, annealing at 59°C for 30 seconds, and extension at 72°C for 30 seconds, with a final extension at 72°C for 10 minutes. All amplicons were separated on a 2% agarose gel in 0.5% Tris-borate-EDTA buffer.

Testing for *Xylella fastidiosa* was conducted as described in another work [42]. qPCR was comprised of forward primer (CACGGCTGGTAACGGAAGA) and reverse primer (GGGTTGCGTGGTGAAATCAAG) 300nM each with 100nM FAM-linked probe (TCGCATCCCGTGGCTCAGTCC). The thermo-cycling conditions were as follows: 50°C for 2 minutes and 94°C for 2 minutes, followed by 40 cycles of 94°C for 10 seconds and 62°C for 40 seconds.

### Bioinformatic analysis

Raw sequence reads were demultiplexed using bcl2fastq allowing for a single mismatch to the expected index combinations and deposited on NCBI’s SRA (BioProject PRJNA1148691). Demultiplexed reads were then trimmed of PCR primer sequences using BBDuK v38 [43], retaining only sequences with primers present in the forward and reverse reads. Sequence quality profiles were then used to remove reads with more than one expected error [44], or those containing ambiguous ‘N’ bases. Due to the natural length variability in the 16S amplicons, a minimum read length of 50bp was applied with no truncation or maximum length filtering. Filtered sequences were dereplicated and denoised into amplicon sequence variants (ASVs) using DADA2 v1.26 [45], with the error model estimated separately for each amplicon, then chimeras were detected and removed de-novo using the removeBimeraDenovo function in DADA2. Taxonomy was assigned to the filtered ASVs using the IDTAXA algorithm [46] implemented in the DECIPHER v2.26 R package, using a separate reference database for each target marker. The ASVs were taxonomically assigned using the Genome Taxonomy Database v202 [47]. Species-level taxonomic assignments with IDTAXA were only accepted if they surpassed a bootstrap confidence threshold of 60% or above, otherwise the ASV was classified to a higher taxonomic rank for which this bootstrap criterion could be achieved. Finally, the ASVs with less than 10 reads were discarded from the dataset before generating a phylogenetic tree using the package phangorn v2.11.1 [48], with the Neighbour Joining algorithm and the GTR model.

### Statistical analysis

**Fig 2:**
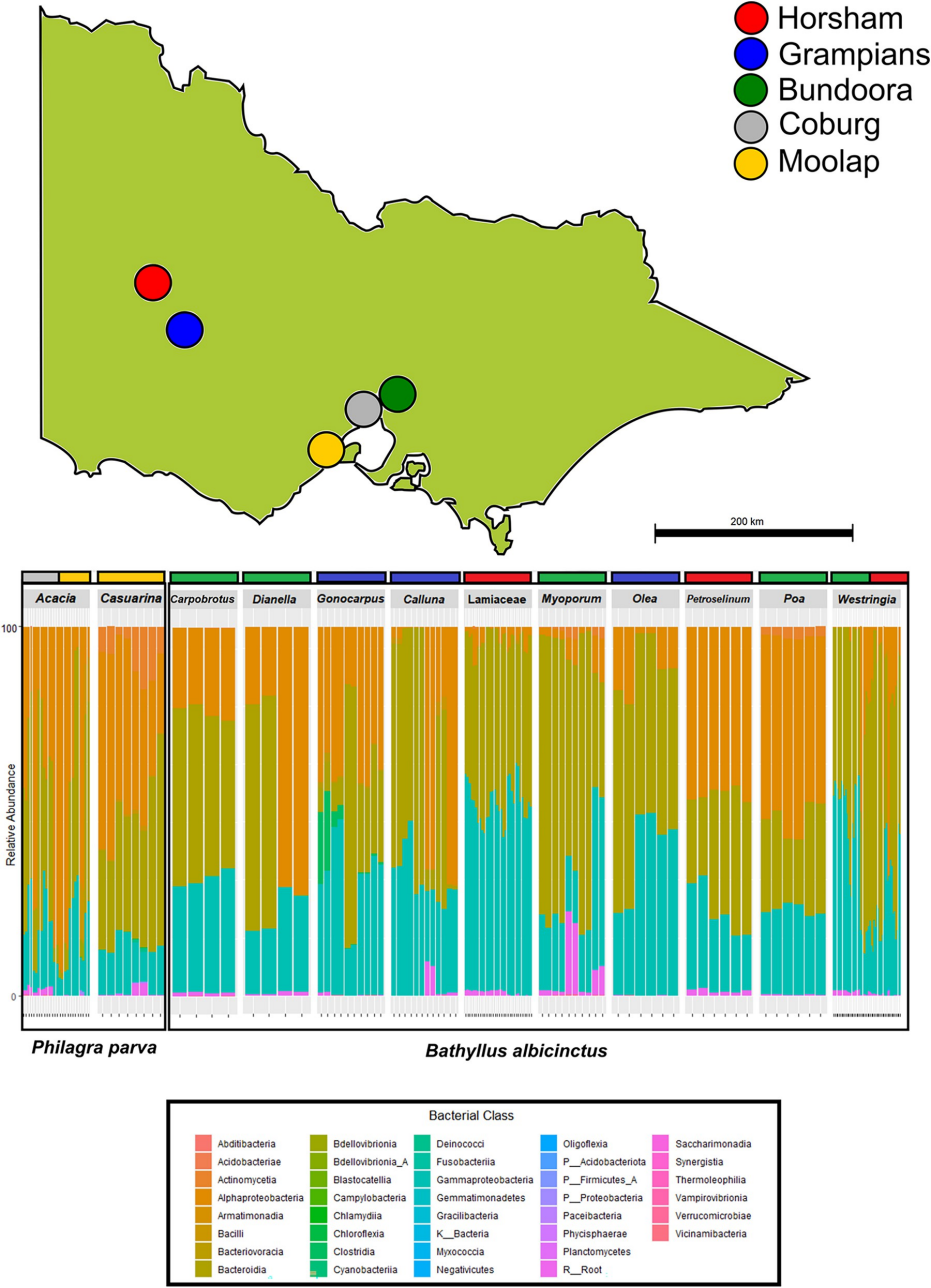
Relative abundance of bacterial classes across *Bathyllus albicinctus* and *Philagra parva*. The plot highlights the diversity of bacterial classes recorded across the 87 samples collected on 12 host plants, from five locations. Locations are colour coded.

All bioinformatic and statistical analyses were conducted within the R v4.3.1 statistical computing environment [49] and RStudio v4.2.2 following the pipeline presented elsewhere [38,39], using amplicon sequence variants (ASVs) as the diversity unit (Fig 2).

**Fig 3:**
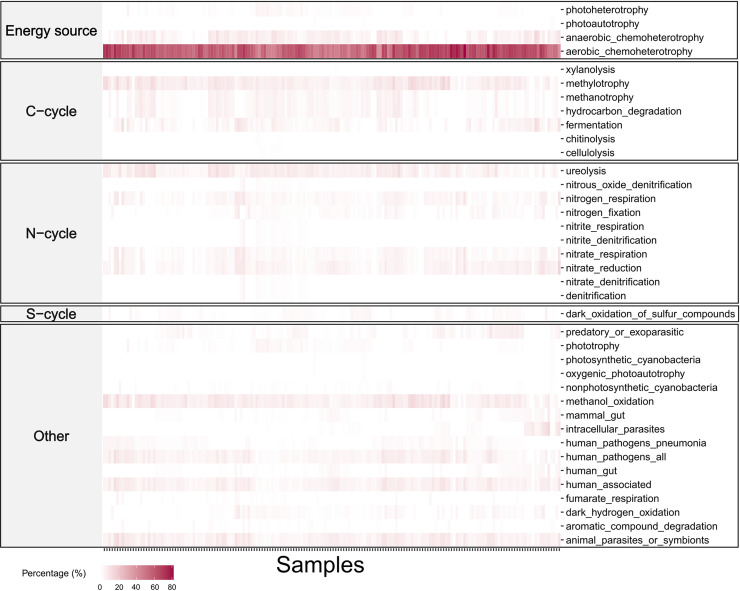
Heat map of functional groups identified for the ASVs of each sample. The main categories (on the left) include different functional groups (on the right). The heat map shows aerobic chemoheterotrophy as the main function across most of the samples analysed in this study.

Bacterial ASVs were assigned to ecological groups utilising the Functional Annotation of Prokaryotic Taxa (FAPROTAX) database v1.2.7 [50], following the analysis presented in a similar work [51], and using the R package microeco v1.6.0 [52] (Fig 3). FAPROTAX is a database which enables bacterial ASVs to be matched to metabolic or ecologically relevant groups by using information collated from published literature.

**Fig 4:**
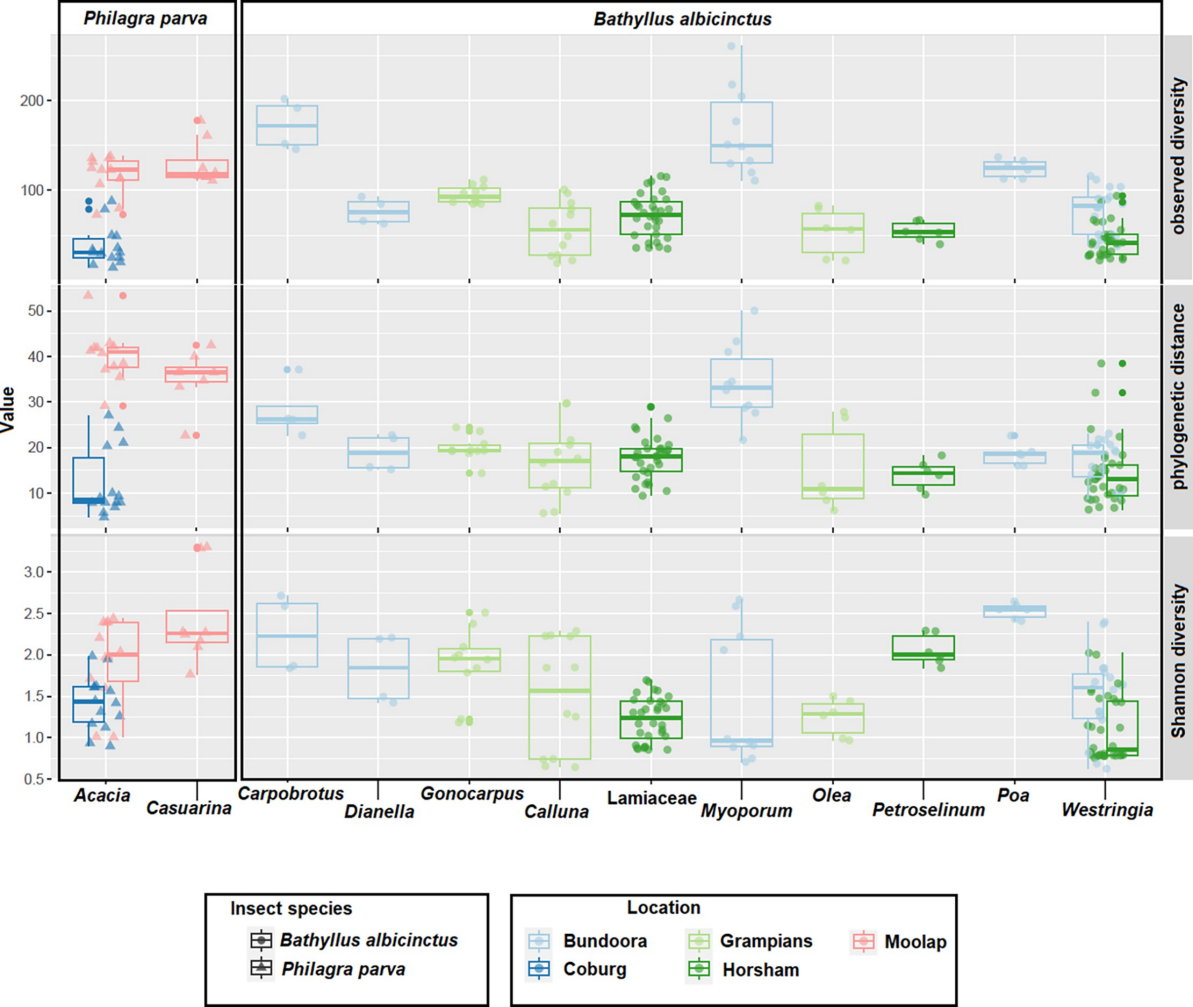
Bacterial α-diversity (observed bacterial species) across the two spittlebug species (*Bathyllus albicinctus* and *Philagra parva*). The figure shows the 12 host plants and the five collection localities (colour-coded). For this plot, α-diversity was measured using observed diversity (number of ASVs).

For α-diversity measures (Fig 4), we used three complementary metrics to account for phylogenetic distance (phylogenetic distance–pd; [53]), abundance (Shannon diversity; [54]), and species presence-absence (observed richness). Observed richness and Shannon diversity were calculated using the phyloseq v1.44 R package [55], and phylogenetic diversity was calculated using the picante v1.8.2 R package [56]. We then used α-diversity to compare mean observed diversity and standard error across all host plants.

**Fig 5:**
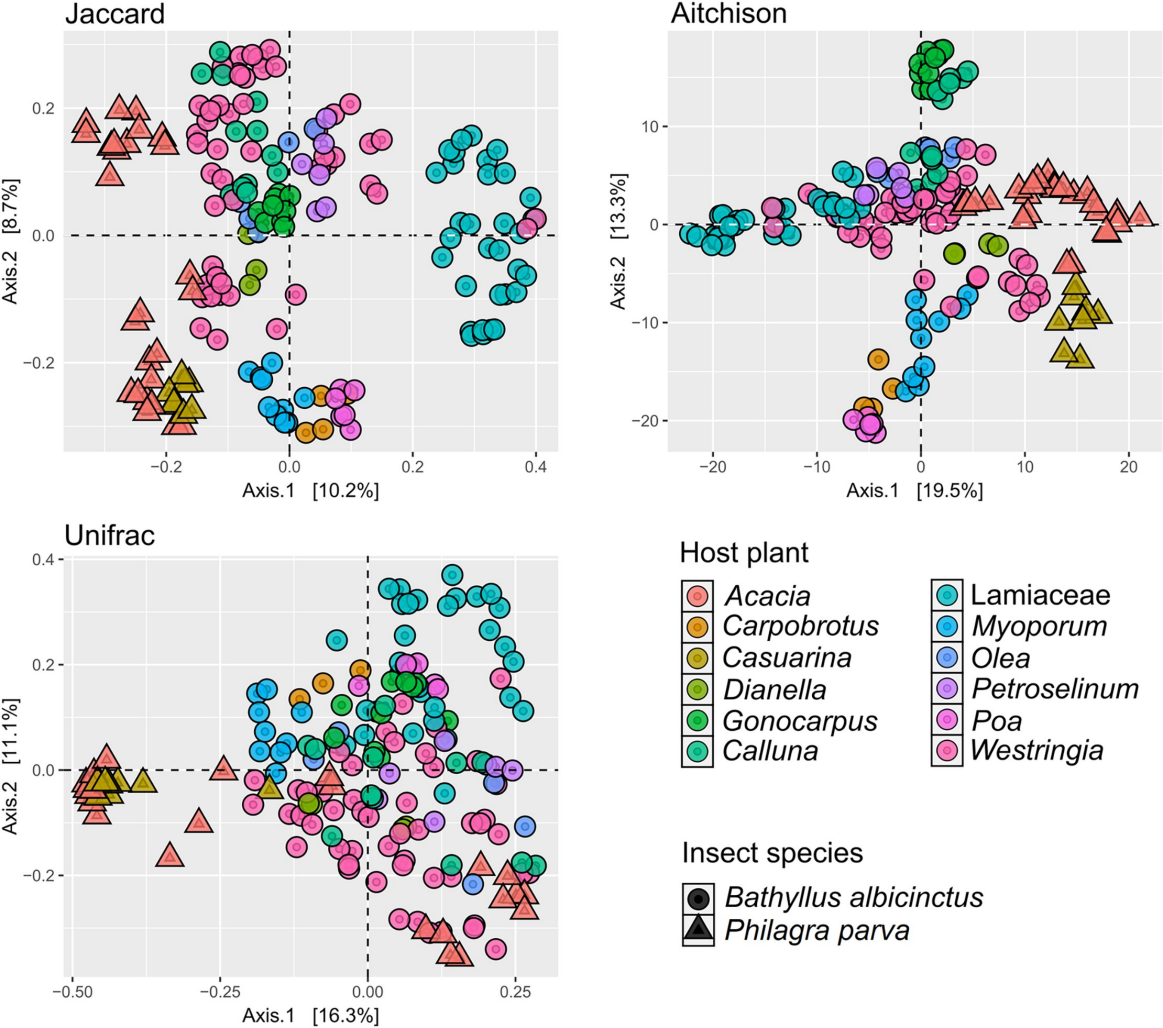
Principal coordinate analysis (PCoA) of the bacterial β-diversity recorded from all samples analysed for this work. The plot shows differences in the β-diversity harboured by insects belonging to different species (*Bathyllus albicinctus* and *Philagra parva*) as well as by populations of the same species feeding on different host plants.

**Fig 6:**
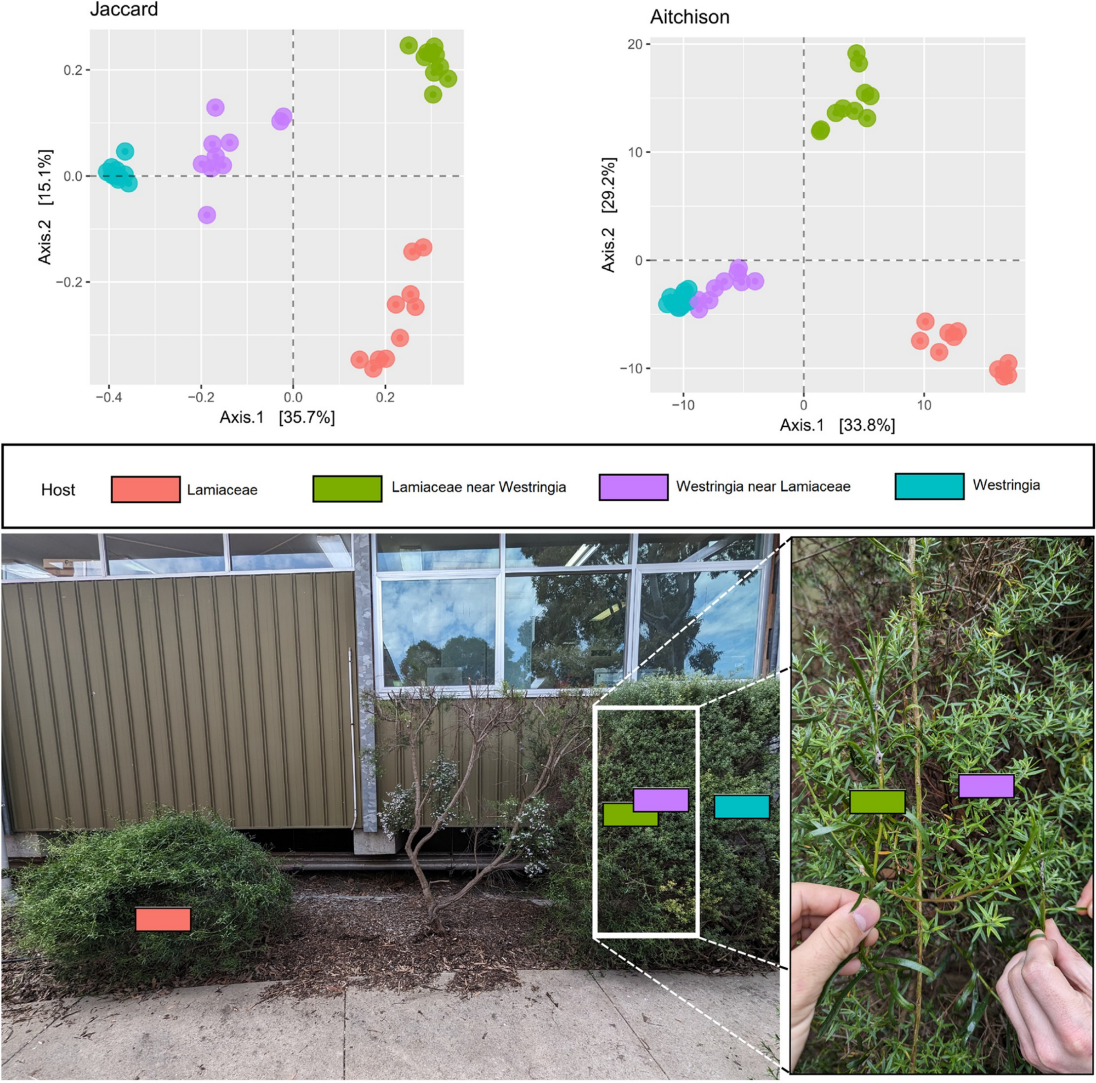
Principal coordinate analysis (PCoA) of the bacterial β-diversity recorded from *Bathyllus albicinctus* specimens collected in Horsham on two plants. The PCoA plot shows differences in the β-diversity harboured by insects feeding on different plants at the same location.

**Fig 7:**
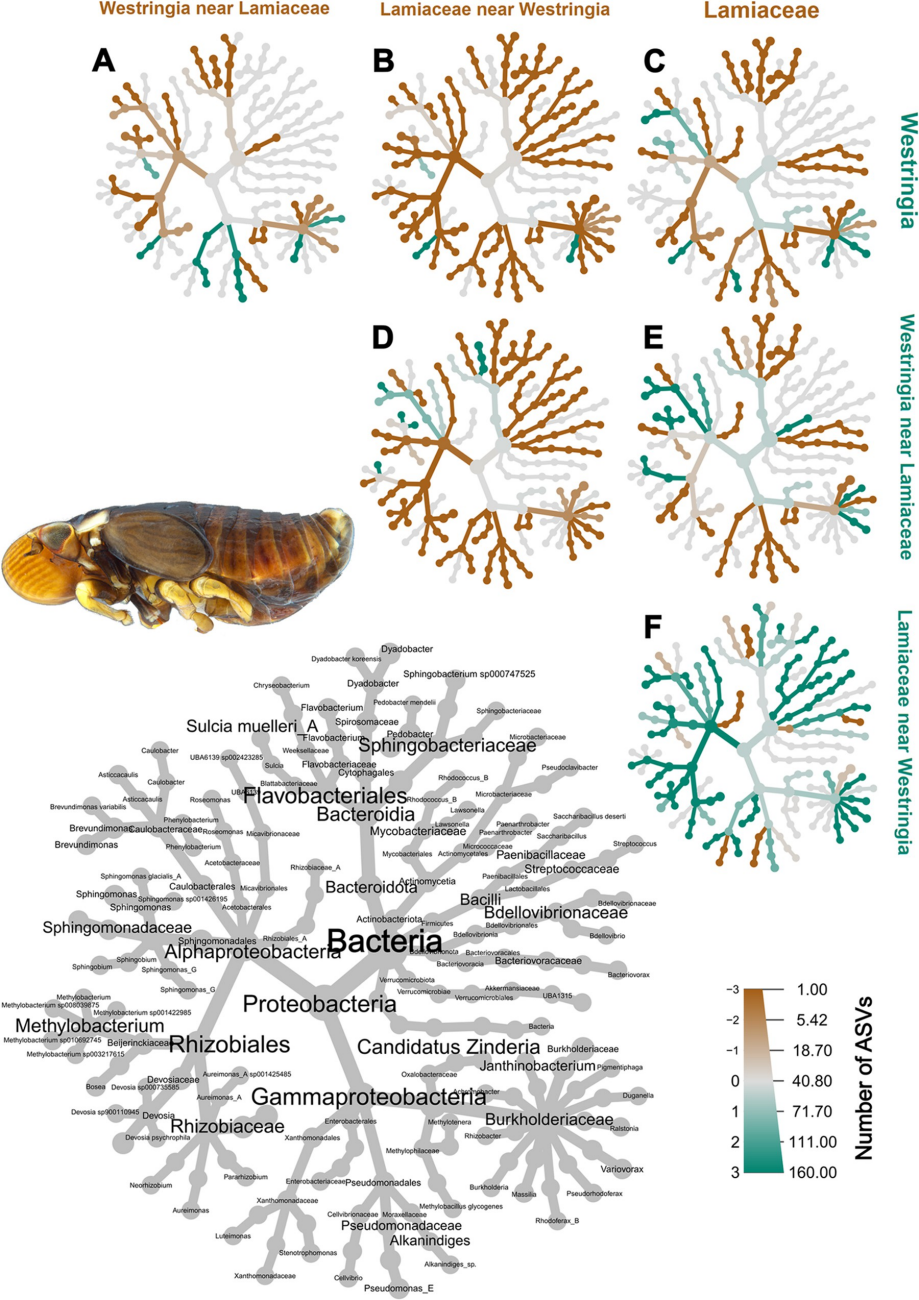
Heat trees comparing the bacterial diversity recorded across different *Bathyllus albicinctus* populations from the Horsham subset, subdivided by host plant. The grey tree on the lower left functions as a key for the unlabelled trees. Each of the smaller trees represent a comparison between two samples, each from a specific plant. A taxon branch coloured brown is more abundant in the sample with the brown label, and a taxon branch coloured green is more abundant in the sample with the green label. A nymph of *B*. *albicinctus* is pictured in the photo. The figure was generated using the R package metacoder [62].

For β-diversity analysis (Figs 5 and 6), we used three distance metrics (Jaccard, Aitchison and UniFrac). These assessed presence-absence of taxa (Jaccard index; [57]), their relative abundance within a compositional data analysis framework (Aitchison distance; [58]) and phylogenetic divergence as well as relative abundance between samples within a similar compositional framework (UniFrac; [59]). Principal coordinate analysis (PCoA) was used to graphically represent the relationships between samples in multidimensional space using the β-diversity dissimilarity matrices. Finally, we compared β-diversity between different host plants using permutational multivariate analysis of variance (PERMANOVA; [60]) tests using the adonis2 function from the vegan v2.6.4 R package [61]. A heat tree was generated in Fig 7 using the R package metacoder v0.3.5.1 [62].

## Results

### Insect COI diversity

A total of 84 COI sequences were generated for this study and submitted to GenBank with Accession numbers PP946014-PP946097. The COI sequences were used to determine the identity of the species included in this work, with 69 sequences matching records available on public database for *Bathyllus albicinctus*, and 15 sequences belonging to *Philagra parva*, matching unpublished records for this species preserved at the Victorian Agricultural Insect Collection (VAIC).

The COI sequences of both *Bathyllus albicinctus* and *Philagra parva* were used to generate a haplotype network analysis to assess whether genetic variation could be observed across populations on different plants. However, this proved not to be the case (Fig 1). While small genetic variations could be observed, ranging between 0–0.56% for *Bathyllus albicinctus* and between 0–0.18% for *Philagra parva*, these variations did not correspond to collection location or host plant. The intraspecific genetic variation observed for both species was <1%, suggesting all specimens collected across Victoria belonged to the same two species.

### Bacterial 16S diversity

A total of 15,736,749 reads were retained after quality control and filtering. These belonged to 1,284 ASVs, belonging to 39 bacterial classes and 84 orders (Fig 2). All 87 samples could be retained, belonging to two species (*Bathyllus albicinctus* and *Philagra parva*), collected from 12 host plants (Fig 2). The top 12 ASVs recorded here contributed 13,602,517 reads (86.44%) and included symbiotic bacteria, plant growth-promoting bacteria as well as a potential pathogenic taxon (Table 2).

**Table 2:** Top 12 ASVs by number of reads recorded across both spittlebug species. These contributed 13,602,517 reads, 86.44% of the total number of reads.

Class	Order	Family	Species	Reads
Bacteroidia	Flavobacteriales	Blattabacteriaceae	*Ca*. Sulcia muelleri	6,776,261
Gammaproteobacteria	Burkholderiales	Oxalobacteraceae	*Ca*. Zinderia sp.	4,125,708
Alphaproteobacteria	Caulobacterales	Caulobacteraceae	*Brevundimonas* sp.	545,021
Alphaproteobacteria	Rhizobiales	Rhizobiaceae	*Agrobacterium* sp.	486,082
Alphaproteobacteria	Rhizobiales	Rhizobiaceae	*Rhizobium* sp.	433,486
Alphaproteobacteria	Rickettsiales	Anaplasmataceae	*Wolbachia pipientis*	332,017
Gammaproteobacteria	Burkholderiales	Oxalobacteraceae	*Ca*. Zinderia insecticola	217,127
Bacteroidia	Flavobacteriales	Weeksellaceae	*Chryseobacterium* sp.	192,723
Gammaproteobacteria	Burkholderiales	Burkholderiaceae	*Pigmentiphaga* sp.	148,379
Gammaproteobacteria	Pseudomonadales	Moraxellaceae	*Alkanindiges* sp.	142,490
Gammaproteobacteria	Burkholderiales	Burkholderiaceae	*Janthinobacterium* sp.	107,390
Gammaproteobacteria	Burkholderiales	Burkholderiaceae	*Ralstonia* sp.	95,833

Of the 1,284 bacterial ASVs retained for this work, 534 (42%) matched with at least one of the 92 functional groups present in the FAPROTAX database (Fig 3). Across our dataset, most bacterial ASVs matched the function of aerobic chemoheterotrophy (Fig 3), followed by anaerobic chemoheterotrophy, fermentation, methanol oxidation, methylotrophy, nitrate reduction and animal symbiosis/parasitism. Some of the ASVs that did not match, such as *Ca*. Sulcia muelleri, are probably too specialised and are not present in the general FAPROTAX database.

The two ASVs with the highest number of reads across all samples were identified as *Candidatus* Sulcia muelleri (6,776,261 reads, ~43%) and *Candidatus* Zinderia sp. (4,125,708, ~26.2%). The first matched with >99% similarity to other sequences present in GenBank, while the second matched *Ca*. Zinderia insecticola, but only with ~94% similarity. A second ASV of a *Ca*. Zinderia sp. (217,127 reads, 1.4%) matched with 100% similarity *Ca*. Zinderia insecticola sequences.

Other confirmed symbiotic and potentially symbiotic bacteria recorded included a species of *Brevundimonas* (545,021 reads, 3.5%), *Wolbachia pipientis* (332,017 reads, 2.1%), a *Pigmentiphaga* species (148,379 reads, 0.94%), and a species from the genus *Alkanindiges* (142,490 reads, 0.91%).

Another bacterium recorded amongst the top 12 was *Janthinobacterium* (107,390 reads, 0.68%), known to be present in the microbiota of many different environments. On the other hand, some of the bacteria recorded could potentially belong to plant pathogenic groups. These include ASVs from the genera *Rhodococcus*, *Rathayibacter*, *Ralstonia*, *Xanthomonas*. All samples were also PCR tested for the presence of pathogenic *Xylella fastidiosa* and *Ralstonia solanacearum*. This testing using species-specific assays produced negative results for all targets.

### Bacterial composition across populations

- When analysing the bacterial composition across samples several findings can be extrapolated from this work. Firstly, both the insect species and the insect-plant association appear to influence the bacterial composition of our insect samples when taking an α-diversity or a β-diversity approach to analysis (Figs 4–6).
- We used observed α-diversity (number of recorded taxa within a sample) across different populations of *Bathyllus albicinctus* (Fig 4) as an initial proxy to assess the diversity across populations. Observed bacterial diversity in the samples collected from *Carpobrotus* and *Myoporum* appeared considerably higher compared to all other populations. On the other hand, the populations collected from *Westringia* in Horsham had the lowest α-diversity (mean observed diversity ± SE: *Carpobrotus* = 173 ± 14.06, *Myoporum* = 165.5 ± 15.42, *Poa* = 124.5 ± 4.11, *Gonocarpus* = 95.2 ± 3.16, *Westringia* from Bundoora = 77.1 ± 6.04, *Dianella =* 76.75 ± 7.28, Lamiaceae = 72.09 ± 4.28, *Calluna* = 56.92 ± 8.68, *Petroselinum* = 54.33 ± 4.37, *Olea* = 53.67 ± 10.83, *Westringia* from Horsham = 43.59 ± 3.24).

In contrast to *B*. *albicinctus*, the two populations of *Philagra parva* collected from Moolap showed a higher observed α-diversity compared to the population collected from Coburg, even when both populations were collected from the same host plant, *Acacia* (Fig 4) (mean observed diversity ± SE: *Casuarina* from Moolap = 130.12 ± 8.86, *Acacia* from Moolap = 116.67 ± 6.12, *Acacia* from Coburg = 37.79 ± 5.89).

We then used β-diversity (diversity between communities) and the ADONIS test to assess and compare bacterial communities across different populations, using different metrics to account for presence/absence (Jaccard), abundance (Aitchison) and genetic distance (Unifrac). This revealed a significant difference in bacterial community composition among insect populations collected from different host plants and in different locations. In populations of *Bathyllus albicinctus*, host plant species accounted for 38%-51% of the variance in sample composition (Jaccard R^2^ = 0.38, p = 0.001; Aitchison R^2^ = 0.51, p = 0.001; Unifrac R^2^ = 0.35, p = 0.001), and location accounted for 12%-24% of the variance (Jaccard R^2^ = 0.15, p = 0.001; Aitchison R^2^ = 0.24, p = 0.001; Unifrac R^2^ = 0.12, p = 0.001). In populations of *Philagra parva*, host plant species accounted for 11%-29% of the variance in sample composition (Jaccard R^2^ = 0.17, p = 0.001; Aitchison R^2^ = 0.29, p = 0.001; Unifrac R^2^ = 0.11, p = 0.008), and between 17% and 42% of variance was explained by the location (Jaccard R^2^ = 0.27, p = 0.001; Aitchison R^2^ = 0.17, p = 0.001; Unifrac R^2^ = 0.42, p = 0.001).

Since different host plants were often collected at different locations, these results may be correlated. When examining the Jaccard PCoA plot (Fig 5) we observed a clear separation between the β-diversity of the two insect species (*B*. *albicinctus* and *P*. *parva*), but we can also highlight a separate clustering of samples collected from Lamiaceae as well as a clear clustering of the samples collected from the same host plant (e.g., *Myoporum*, *Dianella*, *Carpobrotus*).

To better understand the role played by the host plant in shaping the bacterial diversity of the spittlebugs, we selected a subset of 20 samples, all collected in Horsham at the same time of the year, from two host plants: a *Westringia* sp. and an unidentified Lamiaceae sp. (hereafter referred to as Lamiaceae). This dataset enabled us to eliminate any effect caused by the location and collection time and focus our attention on the role of the host plant.

The 20 specimens analysed were collected from four different groups: five were collected from a Lamiaceae plant (Fig 6, left), five were collected from a *Westringia* (Fig 6, right), five were collected from a plant of the same Lamiaceae species, but growing intertwined with the *Westringia* (Fig 6, inset), and five from the *Westringia* with which this Lamiaceae was intertwined (Fig 6, inset). When examining both the Jaccard and the Aitchison PCoA plots, we could clearly observe the bacterial communities of the insects collected from the different groups, with the two groups from Lamiaceae clearly separate from the two groups from *Westringia* (Fig 6).

When comparing the bacterial species between the *Westringia* populations and the Lamiaceae populations (Fig 7), some taxa appeared to be equally present across both host plants. This was the case for both previously described symbionts of spittlebugs (*Ca*. Sulcia muelleri and *Ca*. Zinderia). On the other hand, some taxa appeared to be associated with either one or the other plant (Fig 7). For example, *Brevundimonas*, *Neorhizobium*, and *Massilia* species appeared to be consistently associated with *Westringia* (Fig 7). Amongst the bacteria associated more consistently with Lamiaceae, we recorded *Sphingobacterium*, *Alkanindiges*, *Dyadobacter*, Burkholderiaceae species, and *Devosia* (Fig 7).

## Discussion

### Assessing the impact of insect genetic diversity on bacterial communities

Bacterial communities associated with insects have often been found to be strictly associated with the host’s systematics, with closely related insect species showing more similar bacterial communities, a phenomenon referred to as ‘phylosymbiosis’ [63,64]. This has been shown to be the case for other hemipteran insects, such as psyllids [36], and it is often considered an evolutionary mechanism allowing insects to specialise in feeding from their host plant. To determine whether the insect genetic diversity played a role in shaping the bacterial communities observed in this study, we compared the insect COI gene across all specimens analysed in this work.

The bacterial communities of *Bathyllus albicinctus* clearly separated from those of *Philagra parva*, suggesting that different species may harbour different bacterial communities. While we included only two Australian spittlebug species in this work, and it is therefore impossible to determine phylosymbiosis, these preliminary results do not exclude that some degree of phylosymbiosis may be present in Australian spittlebugs. To confirm phylosymbiosis, further studies should obtain phylogenetic data from Australian spittlebugs and compare it with the insects’ microbial communities to understand whether the “microbial community relationships recapitulate the phylogeny of their host” [65].

The primary aim of this study, however, was to assess if bacterial communities could vary between different insect populations hosted across different host plants. To explore this, the COI sequences of both *Bathyllus albicinctus* and *Philagra parva* were used to generate a haplotype network analysis to assess whether genetic variation could be observed across populations on different plants. This may provide an explanation for differences in bacterial communities associated with insect genetic variation. This expectation, however, was not statistically supported (Fig 1). While small genetic variations could be observed, these variations did not correspond to collection location or host plant. The intraspecific genetic variation observed within both species was <1%, confirming that all specimens collected across Victoria belonged to the same two species.

### Bacterial diversity in *Bathyllus albicinctus* and *Philagra parva*

We recorded the two long-term obligate symbiotic bacteria known to be hosted in spittlebugs: *Ca*. Zinderia insecticola and *Ca*. Sulcia muelleri. Both bacteria were previously recorded in spittlebugs [24] but had never been recorded in *Bathyllus albicinctus* or *Philagra parva*.

Interestingly, this highlights a potentially important difference between the bacterial microbiome of these Australian spittlebugs and *Philaenus spumarius*, the main cercopoid vector of the plant pathogen *Xylella fastidiosa*. In fact, in *P*. *spumarius*, the symbiont *Ca*. Zinderia insecticola has been found to be replaced by a *Sodalis-*like symbiont [25]. The symbiont swap from *Ca*. Zinderia to a *Sodalis* species has been hypothesised to favour host functionality, as free-living bacteria possess many more metabolic capabilities than anciently associated lineages of obligate symbionts [26].

Another symbiotic bacterium recorded in this work was *Wolbachia pipientis*, a maternally transmitted, obligate intracellular bacterium that infects a great number of species of arthropods and nematodes [66]. Similarly, other bacteria we have recorded may play a symbiotic role, although this role is yet to be demonstrated. This may be the case for *Pigmentiphaga*, a genus of bacteria from the family Burkholderiaceae, that has previously been found on human skin, floral nectar, tree sap, stream sediment and soil, as well as in association with amphibian skin, suggesting it may have a beneficial role in protection from pathogenic bacteria [67].

The genus *Alkanindiges* includes bacteria known to contain obligate alkane degraders, implicated in biological foaming in activated sludge systems [68]. These bacteria have been hypothesised to play a role in foam formation or stabilization in waste-water treatment plants, and their presence here may suggest they play the same role in the formation of watery-foam from spittlebug excrement. Interestingly, while no *Alkanindiges* were previously reported in association with spittlebugs, the genus *Brevundimonas* has recently been recorded in association with spittlebug foam and has been hypothesised to play a role in the insect defensive system [27]. Interestingly, the chemical composition of the foam excreted by the juveniles of *Aphrophora alni* (Aphrophoridae) was discovered to primarily consist of fucose, a sugar characteristic of carbohydrate polymers produced by marine bacteria, algae and fungi [69]. Szterk and colleagues even suggested that the foam could be produced by symbiotic bacteria, similar to the fucoidan-secreting bacteria present in brown seaweed [69,70]. The results we obtained indicate that *Alkanindiges-*like bacteria may play a symbiotic role in spittlebugs, and potentially a role in the production of the foam that protects spittlebug nymphs.

On the other hand, most of the bacteria recorded are almost certainly linked to the host plant, as is the case for *Agrobacterium* and *Rhizobium*, two plant growth-promoting rhizobacteria (PGPR), known to facilitate plant growth [71,72].

### Bacterial communities of *B*. *albicinctus* are shaped by host plant associations

The populations of both spittlebug species examined showed differences in bacterial communities associated with host plant species. Due to the limited sampling of *Philagra parva*, we focused our discussion on *Bathyllus albicinctus*, the bacterial community of which differed significantly across populations. Such variation was notably due to the insect-host plant associations rather than to geographic location. While we had limited samples from some of the host plants (i.e., *Carpobrotus*, *Dianella*), and we could collect specimens from the same plant but in different locations only on a limited number of occasions, our dataset was robust enough to assess different host plants from the same location. Indeed, bacterial community composition was found to be extremely variable from insect populations collected from different hosts in the same locality at the same time. This may be due to several factors, including bacterial transmission from the plant to the insect during feeding, as well as to the presence of environmental bacteria on and around the insects, especially during their relatively sedentary nymphal stage. Since this study examined the overall bacterial diversity, and was not limited to the gut content, it is not possible to determine which bacterial species were present inside the insect and which taxa were recorded from the environment. Despite this, the fact that the bacterial communities showed such significant variation across different plants would suggest that even the ‘environmental’ bacteria were circumscribed to a localised distribution that could be defined as a ‘plant environment‘, as opposed to a more general ‘soil environment‘ or ‘air environment‘.

Our study explored, for the first time, the bacterial communities across populations of *Bathyllus albicinctus*, which demonstrated strong variation between different populations. This strong variation, together with the recorded number of bacterial lineages closely related to pathogenic bacteria, may suggest a potential risk for *Bathyllus albicinctus* to vector plant pathogens, should these be present in the plant. In truth, transmission of plant pathogens cannot be determined without further studies, including *in planta* and *in silico* testing (e.g. [73]), or by investigating the underlying biomechanics of pathogen transmission with electrical penetration graphs (EPG) (e.g. [74]), or using scanning electron microscope imaging of the morphological structures involved in the feeding process [75]. Nonetheless, the results obtained suggest spittlebugs harbour a diverse composition of environmental bacteria associated with their host plant. This warrants further studies to effectively model and understand the transmission risks of plant pathogens in Australia linked to native spittlebug species, better preparing Australian disease control efforts for incursions by pathogenic bacteria such as *Xylella fastidiosa*.

## Conclusions

The analysis conducted here showed that both native spittlebug species examined harboured the “traditional” primary symbionts of spittlebugs (*Candidatus* Sulcia and *Ca*. Zinderia), as opposed to the *Sodalis*-like species harboured by *Philaenus*. It would be valuable for future studies to investigate if this is the case for other Australian native species. Indeed, due to the fact *Philaenus spumarius*, a notable cercopoid vector of *Xylella fastidiosa*, is known to have switched one of its primary symbionts, an assessment across Australia and New Zealand would be valuable to determine if any native spittlebugs in these countries have undergone a similar symbiont switch. The identification of any such species could suggest that certain insect species may be more likely to vector similar bacteria, including *Xylella*, and therefore deserves further investigation.
